# Supplementary material for: High cytoplasmic YAP1 expression predicts a poor prognosis in patients with colorectal cancer
Source: PeerJ. 2020 Nov 19;8:e10397. doi: 10.7717/peerj.10397 (PMC7680625; doi:10.7717/peerj.10397)
Supplement: Supplemental Information 4 — Notes:* χ 2 test. ** Mann–Whitney U test (non-parametric). Missing values are excluded for all statistic tests. Abbreviations: CEA, carcinoembryonic antigen; CA19-9, carbohydrate antigen 19-9. [file peerj-08-10397-s004.docx]

**Table S1. The clinicopathological features for the patients at poor+moderate grade or well grade**

| **Characteristics** |  | **Poor+Moderante**  **(n=814)** | **Well**  **(n=95)** | ***P* value^*^** |
| --- | --- | --- | --- | --- |
| **Resected lymph nodes(n(%))** |  |  |  | <0.001 |
| <12 |  | 155(19) | 42(44.2) |  |
| ≥12 |  | 659(81) | 53(55.8) |  |
| **Disease location(n(%))** |  |  |  | 0.211 |
| Rectum |  | 337(41.4) | 33(34.7) |  |
| Colon |  | 477(58.6) | 62(65.3) |  |
| **TNM stage (n(%))** |  |  |  | <0.001^**^ |
| Ⅰ |  | 138(17) | 2(2.1) |  |
| Ⅱ |  | 406(49.9) | 47(49.5) |  |
| Ⅲ |  | 270(33.1) | 46(48.4) |  |
| **Serum CEA(n(%))** |  |  |  | 0.012 |
| <5ng/ml |  | 511(62.8) | 47(49.5) |  |
| ≥5ng/ml |  | 303(37.2) | 48(50.5) |  |
| **Serum CA19-9(n(%))** |  |  |  | 0.032 |
| <37U/ml |  | 701(86.1) | 74(77.9) |  |
| ≥37U/ml |  | 113(13.9) | 21(22.1) |  |

**Notes:** ^*^ χ2 test.

^**^ Mann–Whitney U test (non-parametric). Missing values are excluded for all statistic tests.

**Abbreviations:** CEA, carcinoembryonic antigen; CA19-9, carbohydrate antigen 19-9.
