# Supplementary material for: High cytoplasmic YAP1 expression predicts a poor prognosis in patients with colorectal cancer
Source: PeerJ. 2020 Nov 19;8:e10397. doi: 10.7717/peerj.10397 (PMC7680625; doi:10.7717/peerj.10397)
Supplement: Supplemental Information 5 — Notes:* χ 2 test. ** Student t-test. *** Mann–Whitney U test (non-parametric). Missing values are excluded for all statistic tests. Abbreviations: YAP1, Yes associated protein 1; TNM, tumor-node-metastasis; CEA, carcinoembryonic antigen; CA19-9, carbohydrate antigen 19-9; NCR, Nuclear/Cytoplasmic Ratio. [file peerj-08-10397-s005.docx]

**Table S2. Associations of YAP1 NCR with demographic and clinical variables of 919 CRC patients**

| **Characteristics** | **Total**  **(n=919)** | **YAP1 NCR** | | ***P* Value**^*^ |
| --- | --- | --- | --- | --- |
|  |  | **Low(n=18)** | **High(n=901)** |  |
| **Mean age±SD(year)** | 60.1±12.4 | 64.1±13.4 | 61±12.3 | 0.295^**^ |
| **Sex (n (%))** |  |  |  | 0.069 |
| Men | 549(59.7) | 7(38.9) | 542(60.2) |  |
| Women | 370(40.3) | 11(61.1) | 359(39.8) |  |
| **Disease location (n(%))** |  |  |  | 0.641 |
| Rectum | 512(55.7) | 11(61.1) | 501(55.6) |  |
| Colon | 407(44.3) | 7(38.9) | 400(44.4) |  |
| **Differentiation grade (n(%))** |  |  |  | 0.069^***^ |
| Well | 95(10.3) | 0(0) | 95(10.5) |  |
| Moderately | 779(84.8) | 16(88.9) | 763(84.7) |  |
| Poorly | 35(3.8) | 2(11.1) | 33(3.7) |  |
| Missing | 10(1.1) | 0(0) | 10(1.1) |  |
| **Resected lymph nodes (n(%))** |  |  |  | 0.091 |
| <12 | 201(21.9) | 1(5.6) | 200(22.2) |  |
| ≥12 | 718(78.1) | 17(94.4) | 701(77.8) |  |
| **TNM stage (n(%))** |  |  |  | **0.020^***^** |
| Ⅰ | 140(15.2) | 4(22.2) | 136(16.2) |  |
| Ⅱ | 459(49.9) | 4(22.2) | 455(46.3) |  |
| Ⅲ | 320(34.8) | 10(55.6) | 310(37.4) |  |
| **Chemotherapy (n(%))** |  |  |  | 0.939 |
| Yes | 671(73.0) | 13(72.2) | 658(73) |  |
| No | 248(27.0) | 5(27.8) | 243(27) |  |
| **Serum CEA (n(%))** |  |  |  | 0.159 |
| <5ng/ml | 568(61.8) | 14(77.8) | 554(61.5) |  |
| ≥5ng/ml | 351(38.2) | 4(22.2) | 347(38.5) |  |
| **Serum CA19-9 (n(%))** |  |  |  | 0.700 |
| <37U/ml | 788(85.7) | 16(88.9) | 772(85.7) |  |
| ≥37U/ml | 131(14.3) | 2(11.1) | 129(14.3) |  |

**Notes:** ^*^ χ2 test.

^**^ Student t-test.

^***^ Mann–Whitney U test (non-parametric). Missing values are excluded for all statistic tests.

**Abbreviations:** YAP1, Yes associated protein 1; TNM, tumor-node-metastasis; CEA, carcinoembryonic antigen; CA19-9, carbohydrate antigen 19-9; NCR, Nuclear/Cytoplasmic Ratio.
