# Supplementary material for: High cytoplasmic YAP1 expression predicts a poor prognosis in patients with colorectal cancer
Source: PeerJ. 2020 Nov 19;8:e10397. doi: 10.7717/peerj.10397 (PMC7680625; doi:10.7717/peerj.10397)
Supplement: Supplemental Information 6 — Notes:* χ 2 test. ** Mann–Whitney U test (non-parametric). Missing values are excluded for all statistic tests. Abbreviations: CEA, carcinoembryonic antigen; CA19-9, carbohydrate antigen 19-9. [file peerj-08-10397-s006.docx]

**Table S3. The clinicopathological features for the patients at stage III with or without chemotherapy**

| **Characteristics** |  | **Stage III** | | |
| --- | --- | --- | --- | --- |
|  |  | **Chemo (n=301)** | **No chemo (n=19)** | ***P* value^*^** |
| **Resected lymph nodes(n(%))** |  |  |  | 0.017 |
| <12 |  | 70(23.3) | 0(0) |  |
| ≥12 |  | 231(76.7) | 19(100) |  |
| **Disease location(n(%))** |  |  |  | 0.965 |
| Rectum |  | 160(53.2) | 10(52.6) |  |
| Colon |  | 141(46.8) | 9(47.4) |  |
| **Differentiation Grade(n(%))** |  |  |  | 0.267^**^ |
| Poor |  | 15(5) | 0(0) |  |
| Moderate |  | 236(78.4) | 19(100) |  |
| Well |  | 46(15.3) | 0(0) |  |
| Missing |  | 4(1.3) | 0(0) |  |
| **Serum CEA(n(%))** |  |  |  | 0.730 |
| <5ng/ml |  | 162(53.8) | 11(57.9) |  |
| ≥5ng/ml |  | 139(46.2) | 8(42.1) |  |
| **Serum CA19-9(n(%))** |  |  |  | 0.489 |
| <37U/ml |  | 233(77.4) | 16(84.2) |  |
| ≥37U/ml |  | 68(22.6) | 3(15.8) |  |

**Notes:** ^*^ χ2 test.

^**^ Mann–Whitney U test (non-parametric). Missing values are excluded for all statistic tests.

**Abbreviations:** CEA, carcinoembryonic antigen; CA19-9, carbohydrate antigen 19-9.
