# Supplementary figures and images for: High cytoplasmic YAP1 expression predicts a poor prognosis in patients with colorectal cancer
Source: PeerJ. 2020 Nov 19;8:e10397. doi: 10.7717/peerj.10397 (PMC7680625; doi:10.7717/peerj.10397)

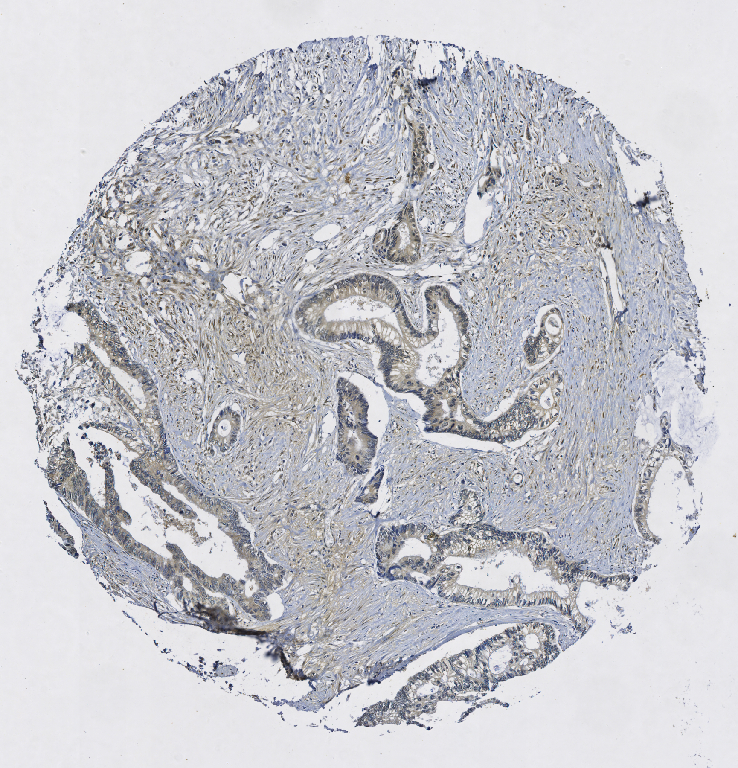

Supplement: Supplemental Information 7 [file peerj-08-10397-s007.zip › raw data/whole field figure of representive IHC staining figure/NCR-H-score 0.6-cancer tissue-whole field~1.tif]

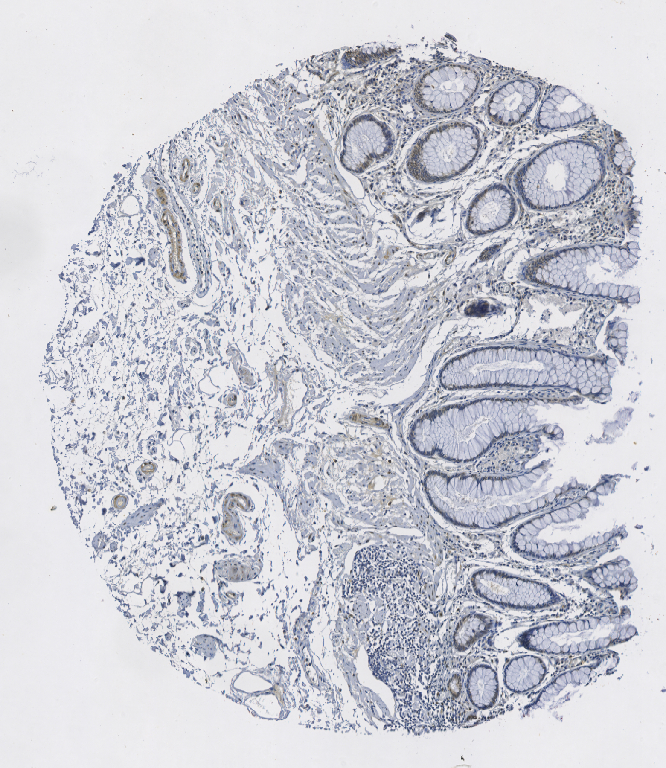

Supplement: Supplemental Information 7 [file peerj-08-10397-s007.zip › raw data/whole field figure of representive IHC staining figure/NCR-H-score 2.5-normal tissue-whole field.tif]

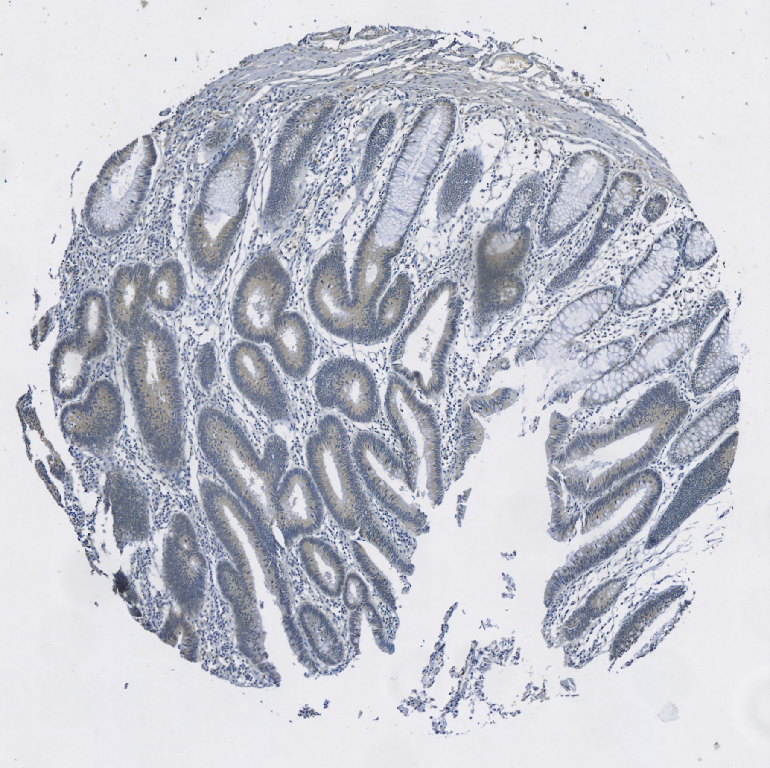

Supplement: Supplemental Information 7 [file peerj-08-10397-s007.zip › raw data/whole field figure of representive IHC staining figure/cytoplasmic-H-score 200-cancer tissue-whole field.tif]

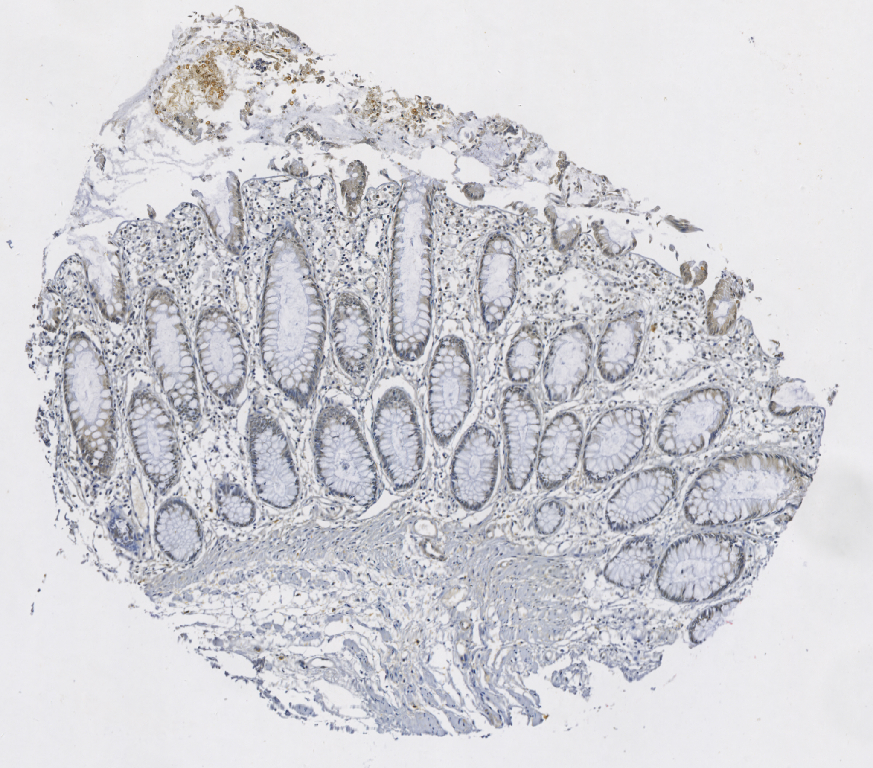

Supplement: Supplemental Information 7 [file peerj-08-10397-s007.zip › raw data/whole field figure of representive IHC staining figure/cytoplasmic-H-score 30-normal tissue-whole field.tif]

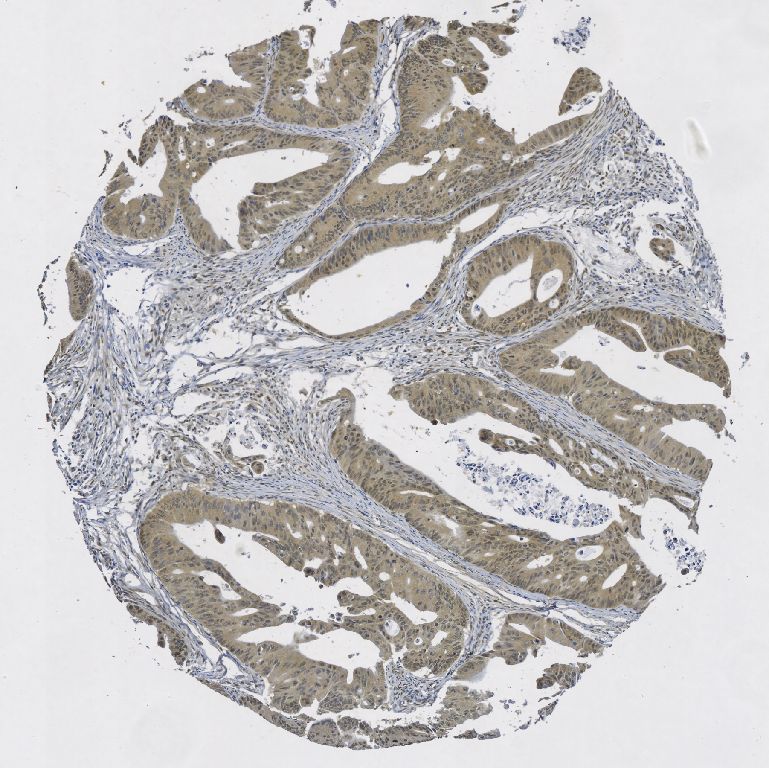

Supplement: Supplemental Information 7 [file peerj-08-10397-s007.zip › raw data/whole field figure of representive IHC staining figure/nuclear-H-score 200-cancer tissue-whole field.tif]

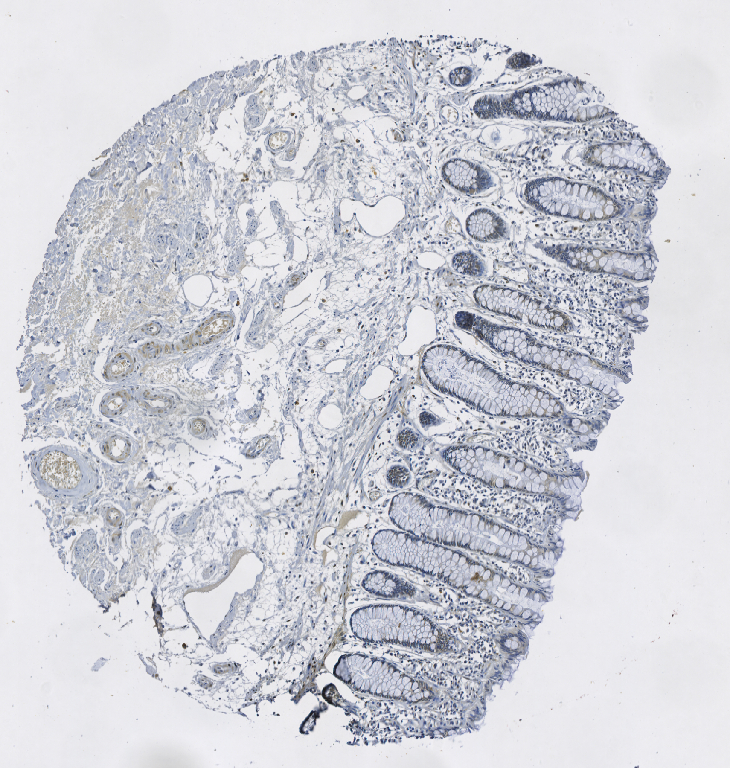

Supplement: Supplemental Information 7 [file peerj-08-10397-s007.zip › raw data/whole field figure of representive IHC staining figure/nuclear-H-score 50-normal tissue-whole field.tif]
